# A New Vaccination Method Based on Phage NgoΦ6 and Its Phagemid Derivatives

**DOI:** 10.3389/fmicb.2022.793205

**Published:** 2022-04-28

**Authors:** Andrzej Piekarowicz, Aneta Kłyż, Daniel C. Stein

**Affiliations:** ^1^Department of Virology, Faculty of Biology, Institute of Microbiology, University of Warsaw, Warsaw, Poland; ^2^Department of Cell Biology and Molecular Genetics, University of Maryland, College Park, College Park, MD, United States

**Keywords:** vaccine, phage display, filamentous phagemid display method without protein fusions, IgG induction, FACS analysis

## Abstract

Phagemid particles based on the *Neisseria gonorrhoeae* filamentous phage NgoΦ6 were used as a vaccine delivery system. We demonstrate that the host proteins incorporated into/associated with these particles can be encoded by chromosomal genes of the host bacterium or from plasmids able to replicate as an autonomous entity in the phagemid host. Phagemid particles were prepared from three types of cells, namely, *Salmonella enterica* ser. Typhimurium [pBSKS::Φ6fm(ST)] containing phagemid genome as an autonomous plasmid, *Haemophilus influenzae* Rd containing phagemid [pBSKS::Φ6fm(Hin)] integrated into the chromosome, and *S. enterica* ser. Typhimurium [pMPMT6::Φ6fm(ST)] containing an additional plasmid, pE1 HCV, encoding the Hepatitis C virus envelope glycoprotein E1. Approximately 200 μg of purified phage particles was used to immunize rabbits. The phagemid particles prepared from these three strains all elicited a large amount of IgG antibodies that were able to recognize bacterial host cells and proteins, as determined by ELISA and FACS analysis. The amount of specific anti-*S. enterica* ser. Typhimurium, anti-*H. influenzae*, and anti-E1 HCV antibodies elicited by vaccination was 170 μg/ml for anti-*Salmonella*, 80 μg/ml for anti-*H. influenzae*, and 65 μg/ml for anti-E1 HCV. Taken in toto, these data suggest that classical phage display methods have underestimated the potential for filamentous phage as a novel immunogen delivery system.

## Introduction

Bacteriophages have been used to make numerous contributions in the development of molecular biology and biotechnology, and in the development of vaccines. In molecular biology, phage display technology has been broadly used to study protein-protein interactions (Henry et al., 2015), library technology, immunotherapy (Hess and Jewell, 2020), antibody phage display technology (Roth et al., 2021), and for general biomedical science applications (Alfaleh et al., 2020). Based on phages, vaccines are classified into three types, namely, phage DNA vaccines, phage display vaccines, and hybrid vaccines (Bao et al., 2019; Zalewska-Piatek and Piatek, 2021). Phage DNA vaccines contain an expression cassette of antigens or mimetics in the phage genome (González-Mora et al., 2020). Phage display vaccines consist of phages that display peptides or proteins fused to phage coat proteins with different peptides or proteins that are expressed as a single protein unit presented on the surface of the phage particle (Stern et al., 2019). The third type of vaccine is a combination of the two types mentioned earlier.

The broad application of phage display technology in the construction of vaccines encounters some difficulties and limitations [see reviews by Bao et al. (2019) and Zalewska-Piatek and Piatek (2021)]. Among them are the necessity of finding a peptide with the correct strong antigenic properties, limitations in the length of peptide that can be fused with the phage protein without influencing phage stability and infection properties, and loss of correct folding of phage protein, fused foreign peptide, or both. While existing phage display methods are based on the formation of a protein fusion between phage structural proteins and foreign peptides, we have developed a phage incorporation method (PhIM) that does not need the formation of protein fusions. This method is based on our previous publications that indicate the following: (1) phage NgoΦ6 can form phagemid particles from any Gram-negative bacterium where the phage genome is cloned into a plasmid that is able to replicate in that organism; (2) phagemid can be transferred into other bacteria by transformation (transfection) or by infection; (3) phagemid replicating in Gram-negative bacteria produce infective phagemid particles; (4) bacteria carrying such phagemid produce phagemid particles that show very strong antigenic properties and elicit antibodies against phage proteins without the use of any adjuvants; and (5) the viral particles of filamentous phages replicating in *Neisseria gonorrhoeae* and phagemids containing cloned NgoΦ6 genome into any type of plasmid contain not only phage-encoded proteins but also proteins encoded by bacterial genome and foreign genes (Piekarowicz et al., 2014, 2016; Kłyż and Piekarowicz, 2018). We showed that bacterial host proteins associated with the phage filament (as identified by mass spectrometry) tended to be one of the predominant outer membrane components of the host strain, plus minor additional host proteins (Piekarowicz et al., 2020). Examples of the proteins include PorB and Opa from *N*. *gonorrhoeae*, flagellin from *S*. *enterica sv*. Typhimurium, and OmpA from *E*. *coli.*

When NgoΦ6 was administrated orally by lysogenic cells of *S. enterica* ser. Typhimurium carrying phagemid pBS::Φ6fm or subcutaneously in the form of purified phage, sera obtained from vaccinated rabbits contained large amounts of IgG and IgA antibodies that were bound by *N. gonorrhoeae* cells and were able to kill the cells, presumably by binding to phages that were being extruded from the bacteria (Piekarowicz et al., 2016; Kłyż and Piekarowicz, 2018). Because the use of filamentous phage/phagemid particles in humans does not show apparent side effects, this indicates their safety. A clinical trial was recently approved by the FDA to allow for intravenous injection of phage to kill bacteria (LaFee and Buschman, 2020). Based on these findings and our previous publications demonstrating the association of host proteins with phage particles, we sought to determine if NgoΦ6 isolated from several bacteria could induce bactericidal responses against the immunizing strain.

## Materials and Methods

### Bacterial Strains, Plasmids, Phages, and Growth Conditions

*Salmonella enterica* sv. Typhimurium χ^3987^ (Galan et al., 1990) obtained from Roy Curtiss III was grown in Luria-Bertani broth (LB) in the presence of diaminopimelic acid (DAP) (100 μg/ml final concentration), *Escherichia coli* Top10 was grown in LB broth, and *H. influenzae* was grown in brain heart infusion broth (BHI) (Difco) supplemented with hemin (10 μg/ml) and nicotinamide adenine dinucleotide (NAD) (2 μg/ml). All bacteria were incubated at 37°C with aeration. Isolation of *S. enterica* ser. Typhimurium, *E. coli*, and *H. influenzae* strains carrying pBSKS::Φ6fm (Hin) was described previously (Piekarowicz et al., 2016). *S. enterica* ser. Typhimurium 3872 (pMPMT6::Φ6fmST) carrying plasmid pE1-HCV [E1 protein (NC004102) virus MycD Tagged ORF clone Origene, United States] was obtained by introduction of the plasmid DNA by electroporation into *E. coli* Top10, purifying the plasmid, and then using the isolated plasmid to transform *S. enterica* ser. Typhimurium #3872 (pMPMT6::Φ6fmST). For convenience, phagemids propagated in *E. coli* are designated as pBSKS::Φ6fm(Ec), in *S. enterica* ser. Typhimurium as pBSKS::Φ6fm(ST), and in *H. influenzae* as pBSKS::Φ6fm(Hin).

### Enzymes and Chemicals

DNA and protein size markers were purchased from Fisher Scientific (Vilno). All chemicals used were of reagent grade or better and were obtained from Sigma-Aldrich (St. Louis, MO, United States), unless otherwise noted. Hepatitis C virus E1 recombinant protein was obtained from ProSci (United States).

### Phage and Phagemid Particle Preparation

To isolate phage and phagemid particles, overnight cultures were diluted 50-fold into 1,000 ml of an appropriate medium and grown overnight with shaking at 37°C. Bacteria were collected by centrifugation (20 min at ∼6,000 RCF). The supernatant was mixed with 1/5 volume of a solution containing 20% polyethylene glycol (PEG-8000) and 2.5 M NaCl and kept at 4°C overnight to precipitate the phage particles. The precipitate was collected by centrifugation (20 min ∼7,500 RCF), dissolved in 4 ml of phosphate-buffered saline (PBS), and centrifuged at 4,000 rpm in an SS34 rotor for 10 min. The phage particles were then purified by sequential centrifugation of the PBS phage suspension at ∼2,000 RCF for 10 min and ∼43,000 RCF for 120 min at 4°C. This procedure was repeated two times. The phagemid particles were further purified and quantified as described previously (Piekarowicz et al., 2020). Phagemid pBSKS::Φ6fm(ST) was additionally purified by CsCl gradient centrifugation as described by Sambrook and Russell (2001).

### Production of Polyclonal Antisera

Samples of purified NgoΦ6 phagemid particles containing ∼200 μg of protein were sent to EUROGENTEC S.A., Liege, Belgium, for immunization of three rabbits according to their standard protocol (ref. no AS-PNOR-3MORAB). In this protocol, phage suspensions were introduced two times subcutaneously at days 0 and 21 in each animal without any adjuvant. Sera were collected at day 0 (before immunization) and at days 21 and 28. To determine the activity, these sera were pooled. All animal work performed at the EUROGENTEC S.A., Liege, Belgium, was carried out in accordance with the 2010/63/EU directive on the protection of animals used for scientific purposes. The protocols were approved under reference CE/SANTE/E/001 by the CER ethical licensing committee.

### Determination of Antibodies Against Phagemid Particles and E1 Protein of HCV Virus by Dot Spot ELISA

Determination of antibodies against phage particles was carried out as described previously (Piekarowicz et al., 2016) with modifications. Bacteria [3 μl of a suspension made of (2 × 10^8^ cells/ml)] diluted in PBS or 10 ng of EP1 protein (NC-004102) in carbonate buffer (50 mM sodium bicarbonate, 0.03 M sodium azide, pH 9.6) were spotted in triplicate onto a nitrocellulose membrane and dried at room temperature. After three washes with 20 ml of PBS buffer, the membranes were blocked with 1% alkaline casein (Sigma) in PBS at 25°C for 1 h. The membranes were then washed three times for 20 min with PBS. Following the washes, different dilutions of sera collected after 0 and 28 days in PBS with 1% alkaline casein were incubated at 25°C overnight. The membranes were washed three times with PBS and incubated with secondary antibodies at room temperature for 1 h [alkaline phosphatase-conjugated goat anti-rabbit IgG (Sigma-Aldrich, United States) diluted in PBS at 1:2,000]. The secondary antibody was removed, and the membranes were washed four times for 15 min each with PBS + 0.1% Tween-20 and once with PBS. Membranes were soaked in 20 ml of detection buffer (AP: 0.1 M Tris-HCl, pH 9.5; 0.1 M NaCl; 5 mM MgCl_2_, pH 9.5) containing 20 μl of NBT BCIP (Sigma, United States) for 30 min at room temperature in darkness. The reaction was stopped by intensive washing of the membrane with distilled water and dried. The amount of protein contained in each spot was visualized on the membrane and quantified using the Quantity One BioRad program. The intensity of each spot was expressed as the increase of the intensity compared with the negative control, where spotting of phagemid or EP1 protein was omitted.

The amount of antibody induced was determined by comparing the optical density of specific anti-*S. enterica* ser. Typhimurium antibodies bound to the spots as presented in a standard curve obtained with known quantities of purified mouse IgG reference antibodies. Standard curves were prepared by the quantitative spot ELISA method. The amount of protein was quantified using GeneTools GBox (Syngen) program and expressed as the intensity of spot versus concentration of IgG protein. Since the measurement range of the ELISA dot was between dilution 2,000 and 5,000, the final determination of IgG concentration in all sera tested was based on the spot intensity values in this range.

### Flow Cytometric Analysis

Flow cytometry was performed according to Price et al. (2007) and described previously in detail (Piekarowicz et al., 2016).

### Western Blotting

For Western blot analysis, proteins were transferred to a positively charged nylon membrane (Roche), blocked with 4% (w/v) non-fat milk in TBS at 25°C for 16 h, and then incubated with various antibodies suspended in TBS at 16°C overnight. After three washes with 20 ml of TBS buffer, the membranes were incubated with secondary antibodies at room temperature for 1 h. Secondary antibody was removed, and the membranes were washed four times for 5 min with TBS + 0.1% Tween-20 and once with TBS. Membranes were then soaked in 20 ml of detection buffer (AP: 0.1 M Tris-HCl, pH 9.5; 0.1 M NaCl; 5 mM MgCl_2_, pH 9.5) containing 20 μl of NBT BCIP for 30 min at room temperature in darkness. The reaction was stopped by washing the membrane with distilled water and dried. The following primary antibodies were used during studies: (1) (DYKDDDK) monoclonal antibodies (FG4R; Thermo Scientific), (2) monoclonal anti-E1 HCV protein (Clone BD198, OriGene), (3) antibodies obtained after subcutaneous vaccination of rabbit with phagemid particles pBSKS::Φ6fm(ST), (4) rabbit serum obtained after subcutaneous vaccination with phagemid particles pBSKS::Φ6fm(Hin), and (5) rabbit serum obtained after subcutaneous vaccination with phagemid particles pMPMT6::Φ6fm(ST) propagated in *S. enterica* ser. Typhimurium carrying plasmid encoding E1 HCV protein (all sera were used with 1:500 dilution). The following secondary antibodies were used: secondary mouse monoclonal 2A9 anti-rabbit IgG heavy β chain (alkaline phosphatase; Abcam; 1:2,000 dilution); and secondary goat anti-mouse IgG H&L (alkaline phosphatase; Abcam; 1:4,000 dilution).

### Transformation Protocols

Electrocompetent cells and transformation of *Salmonella* were carried out according to a procedure described previously (Sambrook and Russell, 2001) and stored at −80°C. Transformed bacteria were selected by plating onto LB agar plates containing appropriate antibiotics.

### Statistical Analysis

All statistical analysis was carried out using Student’s *t*-test using Social Science Statistic web service.^1^ One-tailed *P*-values of < 0.05 were considered statistically significant.

## Results

### Host Proteins Associated With Phagemids pBSKS::Φ6fm(ST) Propagated in *Salmonella enterica* Ser. Typhimurium Elicit Antibodies Against Salmonella Cells

**FIGURE 1:**
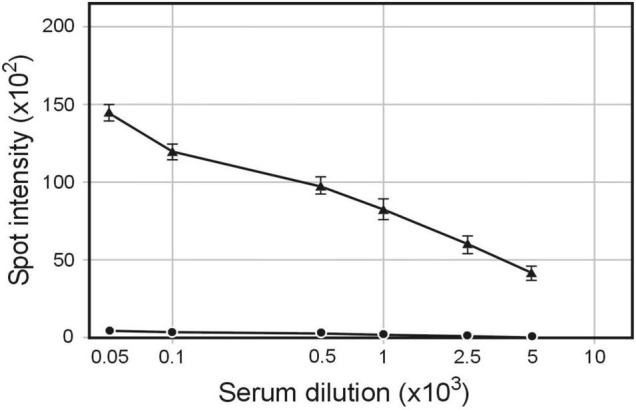
Serum IgG antibodies level elicited by immunization of rabbits with pBSKS::Φ6fm(ST) phagemid particles. Rabbits were immunized subcutaneously with pBSKS::Φ6fm(ST). The sera obtained at day 28 were analyzed by quantitative dot ELISA. *S. enterica* cells (2 × 10^8^ cells/ml) diluted in PBS were spotted on a nitrocellulose strip and allowed to dry. Binding of anti-*S. enterica* was detected with goat anti-rabbit IgG-alkaline phosphatase conjugate. The intensity of the color of each spot was expressed as the change of the spot intensity compared with the negative control where spotting of *S. enterica* was omitted. For each point, four spots were analyzed. Line: 🌑−🌑, 0 day, ▲−▲, day 28.

Our discovery that proteins other than phage filament proteins copurified with bacteriophage particles suggested that these particles could serve as a source of host-cell antigens (Piekarowicz et al., 2020). To test this possibility, we used phagemids propagated in (a) *S. enterica* ser. Typhimurium carrying pBSKS::Φ6fm plasmid, (b) *H. influenzae* Rd carrying pBSKS::Φ6fm phagemid integrated into bacterial chromosome, and (C) strain of *S. enterica* ser. Typhimurium carrying pMPMT6::Φ6 phagemid and plasmid pE1 HCV encoding Hepatitis C virus envelope glycoprotein E1. Approximately 200 μg of protein in the form of phage preparation was used to subcutaneously immunize rabbits without any adjuvant. Quantitative spot ELISA results used for the determination of levels of IgG antibodies specific for *S. enterica* ser. Typhimurium cells elicited by immunization of rabbits with purified pBSKS::Φ6fm(ST) showed significant levels of antibody induction (Figure 1). The above results showed that phagemid pBSKS::Φ6fm(ST) particles induce after subcutaneous delivery a strong immunological response and elicit a large amount of anti-Salmonella antibodies.

**FIGURE 2:**
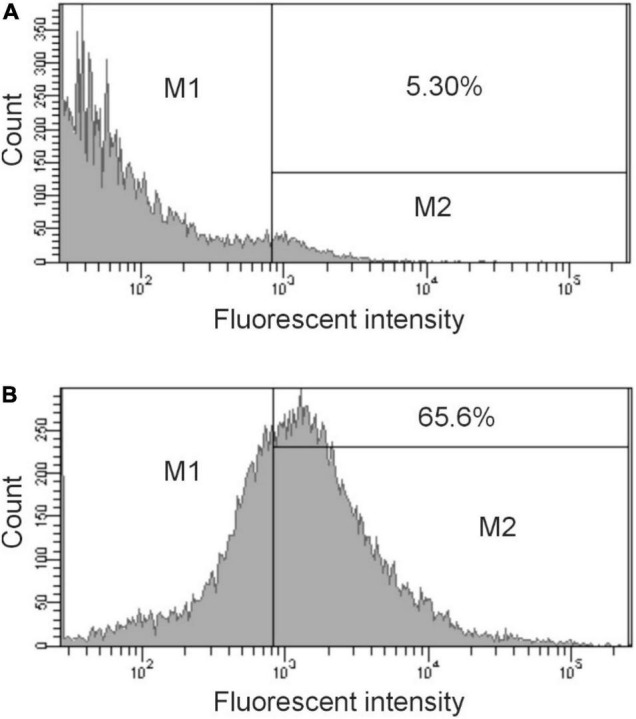
Flow cytometry analysis of antibody binding to *S. enterica* ser. Typhimurium. Cells were treated with preimmunization sera (dilution 1:500) **(A)** or immunized sera (dilution 1:500) obtained after 28 days **(B)** followed by treatment with Cy3 goat anti-rabbit IgG (Life Technologies). The bacteria were analyzed by using FACS Calibur flow cytometer. Data were analyzed with CellQuest. Representative histograms from at least three independent experiments are shown. The bar in the figure represents the gate used to measure binding efficiency. The number in each figure corresponds to the percentage of the population that bound antibody.

The binding activity was further analyzed by flow cytometry. The data in Figure 2B show a significant shift in the binding profile. Using the gating shown in the figure, 65% of the cell population bound significant levels of IgG present in immunized sera (Figure 2B), demonstrating that the elicited antibody was able to bind to intact cells. Preimmunized sera provided minimal binding (Figure 2A).

### Host Proteins Associated With Phagemids pBSKS::Φ6fm(Hin) Elicit Antibodies Against *Haemophilus influenzae* Cells

**FIGURE 3:**
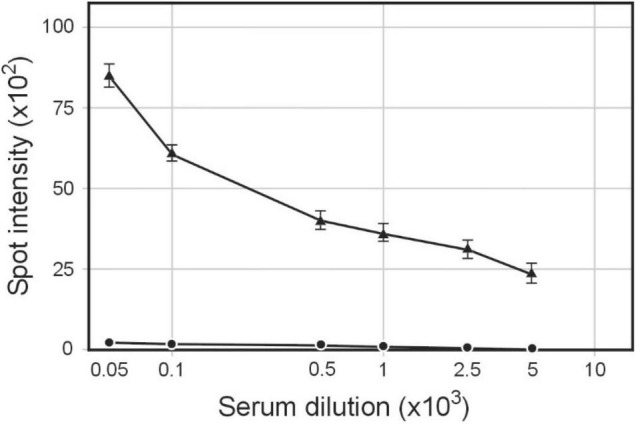
Serum IgG antibodies level elicited by immunization of rabbits with pBSKS::Φ6fm(Hin) phagemid particles. Sera were analyzed by quantitative dot ELISA. *H. influenzae* Rd30 cells (2 × 10^8^ cells/ml) diluted in PBS were spotted on a nitrocellulose strip and allowed to dry. Titers of IgG polyclonal antibodies were collected on day 28 after immunization. The intensity of the color of each spot was expressed as the change of the spot intensity compared with the negative control where spotting was omitted. For each point, four spots were analyzed. anti-*H. influenzae* antibodies bound to the spots. Line: 🌑−🌑, 0 day, ▲−▲, day 28.

**FIGURE 4:**
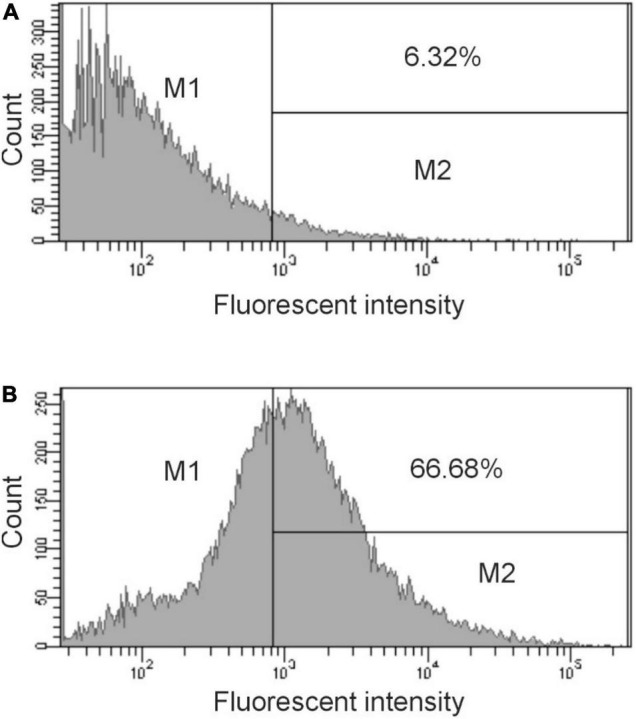
Flow cytometry analysis of antibody binding to *H. influenzae.* Cells were treated with preimmunization sera (dilution 1:500) **(A)** or immunized sera (dilution 1:500) obtained after 28 days **(B)** followed by treatment with Cy3 goat anti-rabbit IgG (Life Technologies). The bacteria were analyzed by using FACS Calibur flow cytometer. Data were analyzed with CellQuest. Representative histograms from three independent experiments are shown. The bar in the figure represents the gate used to measure binding efficiency. The number in each figure corresponds to the percentage of the population that bound antibody.

The DNA sequence of pBSΦ6 is integrated into the chromosome of *H. influenzae* Rd30 cells (Piekarowicz et al., 2016). Lysogenic cells release phagemid particles that contain host cellular proteins (Piekarowicz et al., 2020). We used the same approach as described above to determine if these phagemid particles elicited antibodies against *H. influenzae.* Rabbit sera were obtained from animals immunized with purified pBSKS::Φ6fm(Hin) propagated in *H. influenzae* Rd30. Quantitative spot ELISA results again demonstrated significant levels of IgG antibodies specific for *H. influenzae* cells elicited by immunization of rabbits with purified pBKS::Φ6fm(Hin) (Figure 3). Flow cytometry analysis (Figure 4) shows a significant shift in the binding profile, again demonstrating that the elicited antibody bound intact cells.

### Heterologous Association of Non-host Proteins in Phagemids

**FIGURE 5:**
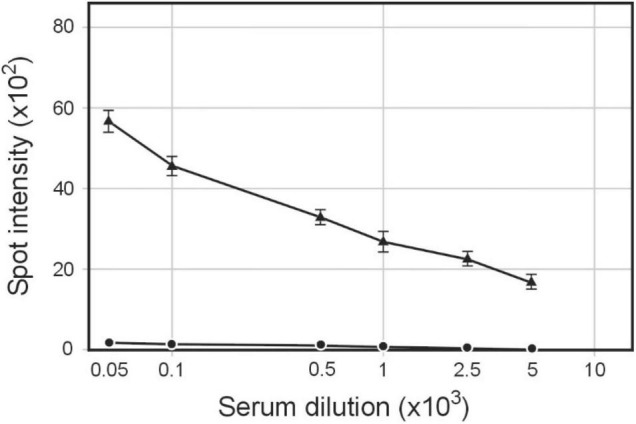
Heterologous expression of non-host proteins in phagemids. Rabbits were immunized with purified pMPMT6::Φ6fm phagemid particles grown in *S. enterica* ser. Typhimurium cells expressing HCV E1. Sera were analyzed by quantitative dot ELISA; 0.1 μg of commercial HCV E1 protein suspended in PBS was spotted on a nitrocellulose strip and allowed to dry. Titers of IgG polyclonal antibodies were collected on day 28 after immunization. The intensity of the color of each spot was expressed as the change of the spot intensity compared with the negative control where spotting of protein was omitted. For each point, 4 spots were analyzed. Line: 🌑−🌑, 0 day, ▲−▲, day 28.

We have previously shown that NgoPhi6 phagemid particles incorporate into/associate with the filament of not only phage structural proteins and host outer membrane proteins but also proteins produced by the expression of foreign genes (Piekarowicz et al., 2020). Purified pMPMT6::Φ6fm phagemid particles grown in *S. enterica* ser. Typhimurium cells expressing the HCV E1 gene incorporate protein E1 into phagemid particles shown by the reactivity of this protein with anti-Myc DDK antibodies. Testing the presence of E1 HCV protein in these phagemid particles showed that the E1 protein is present not only as native 25 kDa protein but also as smaller degradation products. This was also observed for the presence of *Salmonella* flagella protein incorporated into pBSKS::Φ6fm particles propagated in *Salmonella* cells (Piekarowicz et al., 2020). Purified phagemid pMPMT6::Φ6fm particles grown in *S. enterica* ser. Typhimurium cells expressing the HCV E1 protein were used for the immunization of rabbits, and the elicited antibodies were tested for the reactivity with protein E1 HCV. The spot ELISA results used for the determination of levels of IgG-specific HCV E1 protein showed a very high level of antibodies (Figure 5).

### Quantification of Antibody Production

The amount of antibody induced by various vaccine constructs was determined by comparing the optical density of specific anti-target protein antibodies bound to the spots of a standard curve obtained with known quantities of purified mouse IgG reference antibodies. Standard curves were prepared by the quantitative spot ELISA method. Since the measurement range of the ELISA dot was between dilution 2,000 and 5,000, the final determination of IgG concentration in all sera tested was based on the spot intensity values in this range. The amount of specific anti-*S. enterica* ser. Typhimurium, anti-*H. influenzae*, and anti-E1 HCV protein was 170 μg/ml for anti-*Salmonella*, 80 μg/ml for anti-*H. influenzae*, and 65 μg/ml for anti-E1 HCV protein.

### Elicited Antibodies Bind to Host Proteins Present in Bacterial Cells

**FIGURE 6:**
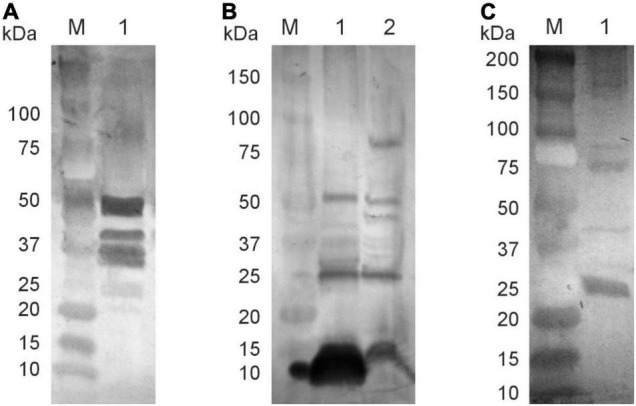
Specificity of elicited antibodies. Phage particles, cellular proteins, and E1 HCV protein were separated on SDS-PAGE gel and subjected to Western blot analysis. Reactivity of rabbit sera obtained after immunization with phagemid pBSKS::Φ6fm(ST) with *S. enterica* ser. Typhimurium proteins (**A**, lane 1); Reactivity of rabbit sera elicited after immunization with phagemid pBSKS::Φ6fm(Hin) with phagemid proteins (**B**, lane 1) and *H. influenzae* Rd30 cell proteins (**B**, lane 2); Reactivity of rabbit sera elicited after immunization with phagemid pBSKS::Φ6fm(ST) propagated in *S. enterica* ser. Typhimurium cells carrying plasmid pE1 HCV with commercial E1 HCV protein (**C**, lane 1). Lanes marked with M indicate molecular weight standards.

The data demonstrate that phagemid particles propagated in either *S. enterica* sr. Typhimurium, *H. influenzae*, or *S. enterica* sr. Typhimurium carrying pE1 plasmid encoding for HSV E1 protein in fact elicit a high level of IgG antibodies. To demonstrate that these antibodies bind to specific cellular proteins present in bacterial cells, several Western blots experiments were performed. We previously showed that flagellin proteins of 50 and 52 kDa molecular size or their degradation or precursor products are the predominant host proteins present in pBSKS::Φ6fm(ST) particles (Piekarowicz et al., 2020). Western blotting was carried out to detect whether they represent also a majority of elicited antibodies in rabbits immunized subcutaneously with pBSKS::Φ6(ST) phagemid particles. The data presented in Figure 6 show reactivity with several proteins, among them proteins in the 50 kDa range (Figure 6A, lane 1), when probed against *S. enterica* ser. Typhimurium cells without phagemids. The outer membrane protein P2 is a predominant host protein associated with pBS::Φ6fm(Hin) phagemid particles (Piekarowicz et al., 2020). Western blotting demonstrated that the phagemid isolated from pBS::Φ6fm(Hin) generated antibodies against both phagemid proteins and several host proteins, one with a mobility similar in mass to the P2 protein (molecular mass of ∼37 kDal) (Figure 6B). Finally, Western blotting demonstrated that phagemids isolated from a strain that also expressed the HCV E1 protein-induced antibody were able to recognize this protein (Figure 6C). From these data, we concluded that phages derived from NgoΦ6 induce immune responses that reflect the host from which they were isolated.

## Discussion

Filamentous phages were described and characterized in the 1960s (see Marvin and Hohn, 1969 for a topical review). Filamentous bacteriophages like Ngoθ6 are members of the genus *Inovirus* in the Inoviridae family. These thread-like viruses have a circular single-stranded DNA genome packaged into a fibrous structure with thousands of copies of coat proteins. During replication, a characteristic feature is that they do not lyse the host (see Hay and Lithgow, 2019 for a recent review). While filamentous phages are known to infect a wide variety of bacterial species (Roux et al., 2019), most of the work utilizing these phages has been based on those that infect *E. coli* (see Stern et al., 2019 for a recent review). This is largely based on the historical role of *E. coli* as a model organism for studying bacteria. The first molecular applications of these phages were for use as cloning vectors for DNA sequencing (Sanger et al., 1980). With the availability of well-developed tools and protocols for engineering phage, the replicative features of *E. coli* filamentous phage have more recently been exploited for use in phage display technology. The first demonstration of filamentous phage as an antigen delivery system was the construction of a fusion protein between the pIII minor coat protein of the bacteriophage f1 and the repeat regions of the circumsporozoite protein gene of *Plasmodium falciparum* (de la Cruz et al., 1988), but their usefulness as antigen delivery systems has largely been limited by the fact that these phages have been limited to the expression of short peptides (de Vries et al., 2021).

There are several dozen vaccines based on filamentous phages that are undergoing testing (see González-Mora et al., 2020 for a recent review), but most of these are anticancer vaccines or vaccines for the prevention of viral infections. This most likely is the result of the fact that fusion between phage structural proteins and peptides to be tested influences the frequency of formation of recombinant phages and their survival. Among all five structural genes of the Inoviridae used in phage display, gp3 is most frequently used, with gp6/gp7/gp9 much less (Jespers et al., 1995; Gao et al., 1999; Carmen and Jermutus, 2002; Ploss and Kuhn, 2011). Because of the complexity of bacterial cell surfaces, it is unlikely that epitope grafting would generate a protective response to these infections.

Our data suggest that the use of Ngoθ6 avoids these obstacles. First, it can be used in a broad spectrum of Gram-negative bacterial strains where phagemids based on phage NgoΦ6 can propagate. The majority of the proteins that we have identified that are incorporated into or associated with phage/phagemid particles belong to outer membrane proteins characterized by the presence of beta-barrel folds (Piekarowicz et al., 2020). While the foreign proteins not only retain their native length, they can also undergo fragmentations, and such fragments are also incorporated (Piekarowicz et al., 2016). As the host proteins that are incorporated into/onto the phage particles are derived from the bacteria used for the propagation of phage, it eliminates the necessity of looking for immunogenic epitopes, increasing the chance that these proteins will retain their native conformation and will act as good antigens and good vaccines.

While the first antibodies produced after vaccination are IgMs, activated B cells subsequently undergo class switching to secrete IgG, among other isotypes. Hence, one hallmark of a good vaccine would be the induction of a strong IgG response. In most cases, increased levels of specific IgG antibodies correlate with increased immunity against the particular pathogen (Klimpel, 1996). Using 200 μg of purified phage/phagemid particles, we were able to generate a strong IgG response after a single boost. These antibodies were reactive with the host cells expressing the phage/phagemid. The data in Figure 6 clearly demonstrate that this response was able to recognize proteins other than those found in the phage genome. That this antibody was able to bind purified HCV protein (Figure 5) demonstrates that the observed reactivity was not due to simple contamination of host proteins with phagemid during the purification process. In toto, our data show that the protein incorporated into/onto phage particles can be derived from heterogeneous genetic elements (plasmids) present in these bacteria. This fact suggests using phagemid particles obtained from such bacteria in the construction of vaccines. Our example is the phagemid particles containing E1 protein of the HCV virus that was expressed from a plasmid.

Before this study can move forward, one needs to assess its limitations. We only immunized three animals and used pooled sera for all of our experiments. This precludes a statistical analysis of potential animal to animal variability. We have previously shown that the gene encoding Orf7 from Ngoθ6, while part of the phage filament, is dispensable (LaFee and Buschman, 2020). This unique property of Ngoθ6 suggests that any gene could replace this coding region, and it would be likely that the new gene would be incorporated into the phage. In this way, one could envision that Ngoθ6 could be used to deliver specific proteins encoded by Gram-positive bacteria or intact viral proteins, demonstrating that this could be a new platform for vaccine delivery. Because the phage particles can be generated from phagemids, it should be possible to engineer the phage so that it would lose its replicative abilities. However, it might be necessary to limit the presence of ß-lactamase or other toxins or toxic proteins found in the host strain, requiring additional manipulation of that host. Modifications in the phage coat could be introduced, which alter the pharmacokinetics of bacteriophages or enhance antigen presentation. IgG antibodies play an important role as an element of immunological memory and therefore a role against reinfections by bacteria and viruses (Wu et al., 2016). In thinking how this filamentous phage can serve as a vaccine platform, one can think about the construction of strain producing phagemid particles in the bacterial strain containing both a phagemid genome and plasmids encoding one of three main membrane proteins of SARS-CoV-2 virus as a potential vaccine against COVID-19. Such a potential vaccine could be constructed very fast in an emergency situation.

## Data Availability Statement

The raw data supporting the conclusions of this article will be made available by the authors, without undue reservation.

## Ethics Statement

The animal study was reviewed and approved by the EUROGENETIC S.A. Liege Belgium.

## Author Contributions

AP: conceptualization, formal analysis, funding acquisition, investigation, methodology, project administration, supervision, validation, and writing – original draft. AK: formal analysis, investigation, methodology, validation, and writing – original draft. DS: conceptualization, funding acquisition, visualization, and writing – review and editing. All authors contributed to the article and approved the submitted version.

## Conflict of Interest

The authors declare that the research was conducted in the absence of any commercial or financial relationships that could be construed as a potential conflict of interest.

## Publisher’s Note

All claims expressed in this article are solely those of the authors and do not necessarily represent those of their affiliated organizations, or those of the publisher, the editors and the reviewers. Any product that may be evaluated in this article, or claim that may be made by its manufacturer, is not guaranteed or endorsed by the publisher.

## Footnotes

1
http://www.socstatistics.com
